# High S100A2 expression in keratinocytes in patients with drug eruption

**DOI:** 10.1038/s41598-021-85009-8

**Published:** 2021-03-09

**Authors:** Manabu Yoshioka, Yu Sawada, Natsuko Saito-Sasaki, Haruna Yoshioka, Kayo Hama, Daisuke Omoto, Shun Ohmori, Etsuko Okada, Motonobu Nakamura

**Affiliations:** Department of Dermatology, University of Occupational and Environmental Health, 1-1 Iseigaoka, Yahatanishi-ku, Kitakyushu, 807-8555 Japan

**Keywords:** Diagnostic markers, Health care

## Abstract

Telaprevir used as a protease inhibitor against hepatitis C virus is frequently associated with cutaneous adverse reactions. To explore a histological biomarker of cutaneous adverse events induced by telaprevir, we systematically searched for genes that were dysregulated by telaprevir in normal human epidermal keratinocytes (NHEKs). Microarray analysis and real-time polymerase chain reaction (PCR) revealed the significant increase in the expression of S100 calcium-binding protein A2 (*S100A2*) gene following treatment of NHEKs with telaprevir. Immunohistochemical analysis demonstrated that the expression of S100A2 was dominant in the spinous layer of the epidermis in patients with telaprevir-mediated severe-type drug eruptions and limited to the basal layer of the epidermis in healthy subjects. Furthermore, S100A2 expression increased after treatment with trichloroethylene and other medications, and the degree of S100A2 expression correlated with the severity of cutaneous adverse events. S100A2 expression also significantly increased in the skin of patients with atopic dermatitis and psoriasis. Taken together, S100A2 is highly expressed in the epidermis under inflammatory conditions and drug eruptions and may serve as a marker for keratinocyte damage in response to any inflammatory or toxic condition.

## Introduction

The peripheral immune system regulates homeostasis of the human body by protecting against various environmental stimuli^1^. The skin is a representative peripheral lymphoid organ located in the outer layer of the human body^2^, and is exposed to external antigens, which may cause cutaneous allergic reactions. Drug eruption is a representative major allergic disease and a life-threatening risk in conditions such as Stevens–Johnson syndrome and toxic epidermal necrolysis^3^. Its diagnosis at an early stage is imperative for clinicians. Although there are some key mediators for disseminated keratinocyte death in Stevens–Johnson syndrome and toxic epidermal necrolysis^4^, the clinical outcome of treatment for severe-type drug eruption remains unsatisfactory. Therefore, we searched for another histological marker in this study.

Telaprevir is a hepatitis C virus (HCV) protease inhibitor that exhibits strong antiviral activities in combination with ribavirin and interferon-α in a previous unsuccessfully treated chronic HCV infection, contributing to a 53% of sustained virologic response^5^. However, approximately 46.8% patients receiving telaprevir, ribavirin, and interferon-α triple therapy developed cutaneous adverse events higher than grade 2 according to Common Terminology Criteria for Adverse Events (CTCAE) as compared to 23.8% patients on ribavirin and interferon-α treatment^6^. Therefore, it is assumed that telaprevir itself might have an unfavorable effect on the exacerbation of cutaneous drug response.

S100 calcium-binding protein A2 (S100A2) is a member of the EF-hand motif family S100^7^. The S100 family comprises more than 20 members of highly conserved acidic calcium-binding proteins. Numerous S100 proteins are known to be overexpressed in cancers, of which S100A2 is thought to primarily exhibit a tumor-suppressive role^8^. S100A2 is mainly expressed in epithelial cells and contributes to various cell functions^9^. However, the expression of S100A2 in cutaneous inflammation in the human body remains unclear.

In this study, we focused on telaprevir as a tool to discover useful markers for drug eruptions because it is known to frequently induce severe drug eruptions. Microarray analysis of keratinocytes after telaprevir treatment revealed that *S100A2* expression increased in the epidermis of patients with drug eruptions. Furthermore, we found S100A2 overexpression in the epidermis of patients with other inflammatory skin diseases, suggesting the role of S100A2 as a marker of keratinocyte damage in response to any inflammatory and toxic condition.

## Material and methods

### Patients

Seven patients with telaprevir-mediated drug eruptions were enrolled in this study. The diagnosis of telaprevir-related drug eruption was made by clinical course and histological examinations. The clinical characteristics were examined by age, sex, duration after telaprevir intake, and results of the lymphocyte stimulation test (LST). The severity of cutaneous adverse events was determined based on the guidelines for the use of telaprevir.

In addition, to precisely evaluate S100A2 expression in the epidermis, we examined 55 cases of mild form of drug eruption (41 cases of macular type and 14 cases of maculopapular type) and 9 cases of severe type of drug eruption (7 cases of Stevens–Johnson syndrome and 2 cases of toxic epidermal necrolysis) and investigated any difference in S100A2 distribution in the skin. S100A2 expression distribution was divided into two types, namely, the basal layer and spinous layer, indicating the extended expression of S100A2 into the spinous layer from the basal layer, and the basal layer suggesting the limited S100A2 expression within the basal layer.

### Histology and immunohistochemistry

Paraffin-embedded skin specimens were prepared using routine methods. The sections were stained with hematoxylin and eosin. Immunohistochemistry was performed as previously described with some modifications^10^. In brief, sections were deparaffinized and hydrated by washing in xylene and treatment with graded alcohol series. To unmask antigens, sections were incubated in 10 mM citric acid (pH 6) (Dako) at 95 °C for 30 min. Sections were then blocked with normal serum for 60 min at room temperature and then treated with primary antibodies (S100A2; Abcam, Cambridge, UK). Samples were washed and incubated for 30 min with secondary antibodies and visualized by staining with 3,3-diaminobenzidine.

### Keratinocyte culture

Normal human epidermal keratinocytes (NHEKs) (neonatal foreskin from Kurabo, Osaka, Japan) were cultured in HuMedia-KG2 medium (Kurabo). After incubation with or without 2 mM trichloroethylene (TCE) (Sigma, St. Louis, MO, USA) for 36 h or 3.96 µg/mL telaprevir for 24 h, the cells were harvested and total RNA immediately extracted. The experiments were performed in triplicates.

### Real-time polymerase chain reaction (PCR)

Real-time quantitative PCR analysis was performed as previously reported with some modifications^11^. In brief, total RNA was extracted from keratinocytes using a TRIzol RNA extraction kit (Invitrogen, Carlsbad, CA, USA). Total RNA was purified using the Qiagen RNeasy Mini Kit (Qiagen, Hilden, Germany) and its A260/A280 ratio was measured with Bio Photometer (Eppendorf, Hamburg, Germany). The purified total RNA was reverse-transcribed into complementary DNA (cDNA) with 1st strand cDNA synthesis kit for RT-PCR (AMV) (Roche Diagnostics, Mannheim, Germany). Real-time quantitative PCR was performed by monitoring the synthesis of dsDNA during the various PCR cycles using assays-on-Demand Gene Expression Assay Mix (Hs00195582_m1 and Hs00266705_g1 respectively, Applied Biosystems, Foster City, CA) with a 7000 system (Applied Biosystems). The data were analyzed with the ΔΔCt method. The expression of genes was normalized to that of the housekeeping glyceraldehyde-3-phosphate dehydrogenase mRNA.

### Microarray

Keratinocytes were treated with telaprevir or TCE, and total RNA was extracted using Trizol reagent (Invitrogen). Total RNA was used to develop an RNA microarray using a hybridization chamber (Takara, Otsu, Japan) and 3D-Gene Scanner 3000 (Toray Industries, Japan). The data were analyzed using 3D-Gene Extraction Software (Toray Industries).

The total RNA was amplified for two types of samples (control and telaprevir-treated or TCE-treated samples) from NHEK cells labeled with Cy3 and Cy5, respectively. Control-Cy3 and telaprevir- or TCE-Cy5-treated samples were applied to the chip, and hybridization was performed. Expression fold change was compared with control samples, and fold changes more than 1.6 were evaluated as significant upregulation or downregulation as compared with the normalized sample value of more than 100 according to the instructions of the 3D-Gene kit analysis. Gene Ontology (GO) enrichment analysis was performed by Metascape to identify the top-level GO biological processes under the stimulation of agents^12^.

### Microarray data analysis

The data on *S100A2* expression in the skin of 33 psoriasis cases and 21 healthy controls as well as 13 atopic dermatitis (AD) cases with lesional skin and 8 healthy controls were analyzed using the public data sets from the National Center for Biotechnology Information (NCBI) Gene Expression Omnibus (GEO) database (GEO accession no. GDS3539 and GDS4491)^13,14^. The expression of *S100A2* gene was shown as an index Z-value. P-values were calculated by the Student’s *t*-test to compare the expression of *S100A2* between healthy subjects and patients with psoriasis or AD. The correlation between the severity of AD (SCORAD) and the Z-value of S100A2 expression was also examined in the lesional skin and non-lesional skin of patients with AD. To identify organs with dominant S100A2 expression, we analyzed a public data set deposited in the GEO database (GEO accession no. GDS3834) and evaluated *S100A2* expression as Z-value^15^.

### Study approval

The study design was approved by the review board of the University of Occupational and Environmental Health. The study was conducted according to the Declaration of Helsinki guidelines. As this was a retrospective cohort study, the opt-out method of obtaining the waiver of an informed consent was adopted based on the approval of the ethics committee’s review board of the University of Occupational and Environmental Health.

## Results

### Clinical characteristics of telaprevir-related drug eruption

As telaprevir frequently induces cutaneous drug eruption, it may exacerbate cutaneous drug reactions. To address this issue, we first analyzed the clinical characteristics of telaprevir-related drug eruption. Seven patients with telaprevir-related drug eruptions visiting our department were enrolled in this study. Table 1 shows the clinical characteristics of telaprevir-related drug eruptions. The skin eruptions spread onto the whole body for three patients but were limited to the extremities in four patients. Severity grade according to telaprevir drug eruption guidelines were as follows: 3 cases were grade 2 and 4 cases were grade 1. Although LST was negative in all five tested individuals, no probable causative new drug other than telaprevir was administered to each patient just before the appearance of eruptions. Moreover, the erythema disappeared after cessation of telaprevir administration, confirming drug eruption by telaprevir.

**Table 1:** Summary of the patients with adverse skin reactions by telaprevir.

Case	Age	Sex	Duration of intake of telaprevir (days)	Distribution of the erythema	Grade	S100A2 expression	LST
1	66	M	3	Hand, foot	1	Basal layer	Negative
2	60	M	3	Arm, leg	1	Basal layer	Negative
3	62	F	4	Thigh	1	Not examined	Negative
4	62	M	25	Whole body	1	From basal layer to spinous layer	Not examined
5	64	F	93	Trunk, Arms	2	From basal layer to spinous layer	Negative
6	47	M	3	Face, trunk, arms	2	From basal layer to spinous layer	Negative
7	51	F	9	Upper arm	1	Basal layer	Not examined

### High expression of S100A2 in keratinocytes under telaprevir

**Figure 1:**
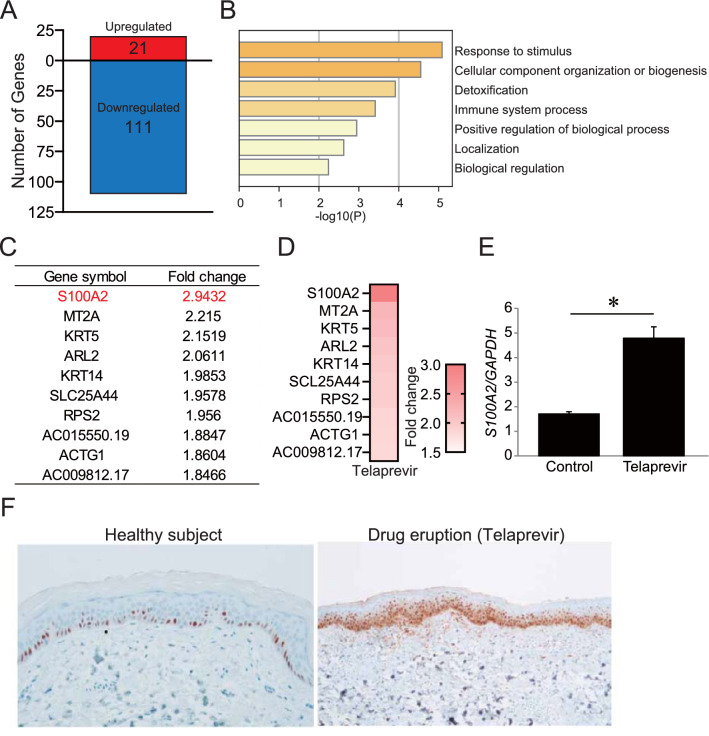
The expression of S100A2 in telaprevir-treated keratinocytes. (**A**) Microarray analysis of the dysregulated genes in keratinocytes after telaprevir treatment. (**B**) GO enrichment analysis of upregulated genes in telaprevir-treated keratinocytes. (**C**,**D**) The list (**C**) and heat map (**D**) of top 10 upregulated genes in keratinocytes by telaprevir. (**E**) *S100A2* gene expression in keratinocytes. (**F**) Immunostaining for S100A2 in the skin. Results are expressed as the mean ± SD. p-values were obtained by the Student’s *t*-test: *p < 0.05.

The skin is the outermost layer of the human body, and keratinocytes form a major cell population on the skin surface. Keratinocytes interact with other cutaneous cells and drive the expression of several cytokines such as thymic stromal lymphopoietin (TSLP) and tumor necrosis factor (TNF)^16,17^. Therefore, we speculated that keratinocyte-derived factors may interact with peripheral blood immune cells, leading to the development of drug eruptions after telaprevir treatment owing to the negative results in LST. To address this issue, we examined the gene expression in keratinocytes by microarray analysis and explored the candidate genes involved in the amplification of the inflammatory response to telaprevir. Unexpectedly, TSLP and TNF expression did not increase in telaprevir-treated keratinocytes in this microarray analysis. Therefore, we comprehensively explored the possible underlying factors that activate cutaneous inflammation. In keratinocytes treated with telaprevir, 21 upregulated and 111 downregulated genes were identified (Fig. 1A). GO analysis of the set of upregulated genes by telaprevir revealed enrichment of inflammatory responses such as response to stimuli, immune system process, and positive regulation of biological processes (Fig. 1B). Figure 1C,D shows the top 10 upregulated genes, of which *S100A2* was the most upregulated gene after telaprevir treatment. Consistently, qPCR validation showed a significant increase in *S100A2* expression in keratinocytes after telaprevir treatment (Fig. 1E). Although S100A2 expression mainly increased in the basal layer of the epidermis in healthy subjects, an increase was also noted in the spinous layer in severe-type drug eruptions owing to telaprevir (Fig. 1F). These findings indicate that S100A2 in keratinocytes may be involved in drug eruption.

### S100A2 expression increased in keratinocytes with TCE stimulation

**Figure 2:**
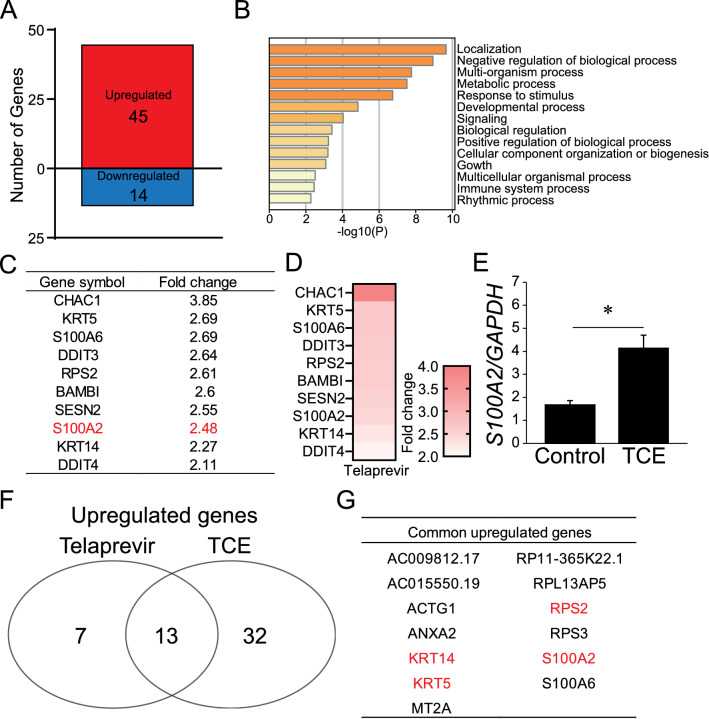
The expression of S100A2 in TCE-treated keratinocytes. (**A**) Microarray analysis of the dysregulated genes in keratinocytes after TCE treatment. (**B**) GO enrichment analysis of upregulated genes in TCE-treated keratinocytes. (**C**,**D**) The list (**C**) and heat map (**D**) of top 10 upregulated genes in keratinocytes by TCE. (**E**) The number of common upregulated genes in telaprevir- and TCE-treated keratinocytes. (**F**) The list of common upregulated genes in telaprevir- and TCE-treated keratinocytes. Four common upregulated genes within the top 10 lists in both telaprevir and TCE groups are highlighted in red. Results are expressed as the mean ± SD. p-values were obtained by the Student’s *t*-test: *p < 0.05.

As patients who develop drug eruptions are prescribed suitable medications, it is difficult to exclude the influence of these medications on cutaneous drug responses. Indeed, some medications have been shown to exert anti-inflammatory effects against immune cells^18,19^. Therefore, it may be necessary to evaluate the effect on S100A2 upregulation in keratinocytes using a non-medication agent. TCE, a representative material, is a chloric organic compound frequently used in industry and known to cause occupational chemical-induced skin toxicity in healthy subjects, including Stevens-Johnson syndrome and hypersensitivity^20–26^. To clarify the fluctuation in the gene profile following TCE stimulation, we performed microarray analysis for genes in keratinocytes treated with TCE. There were 45 upregulated and 14 downregulated genes under TCE stimulation (Fig. 2A). GO analysis of the set of TCE-mediated upregulated genes showed the enrichment of response to stimuli, positive regulation of biological process, and immune system process (Fig. 2B) along with negative regulation of biological process. Figure 2C,D shows the top 10 upregulated genes, and S100A2 was listed as the eighth most upregulated gene after TCE treatment. Consistently, the validation of qPCR analysis showed a significant increase in *S100A2* expression in keratinocytes after TCE treatment (Fig. 2E).

Next, we explored the commonly upregulated genes between telaprevir and TCE, and identified 13 candidates (Fig. 2F). Figure 2G shows that there were four common upregulated genes within the top 10 candidates (Figs. 1C, 2C). Excluding *S100A2*, *KRT5*, *KRT14*, and *RPS2* are genes encoding cellular component proteins^27,28^. In addition, *S100A2* was the most highly upregulated gene in keratinocytes following telaprevir treatment. Therefore, we speculate that *S100A2* might be involved in the pathogenesis of cutaneous drug response.

### S100A2 expression in patients with drug eruption caused by other causative agents

**Figure 3:**
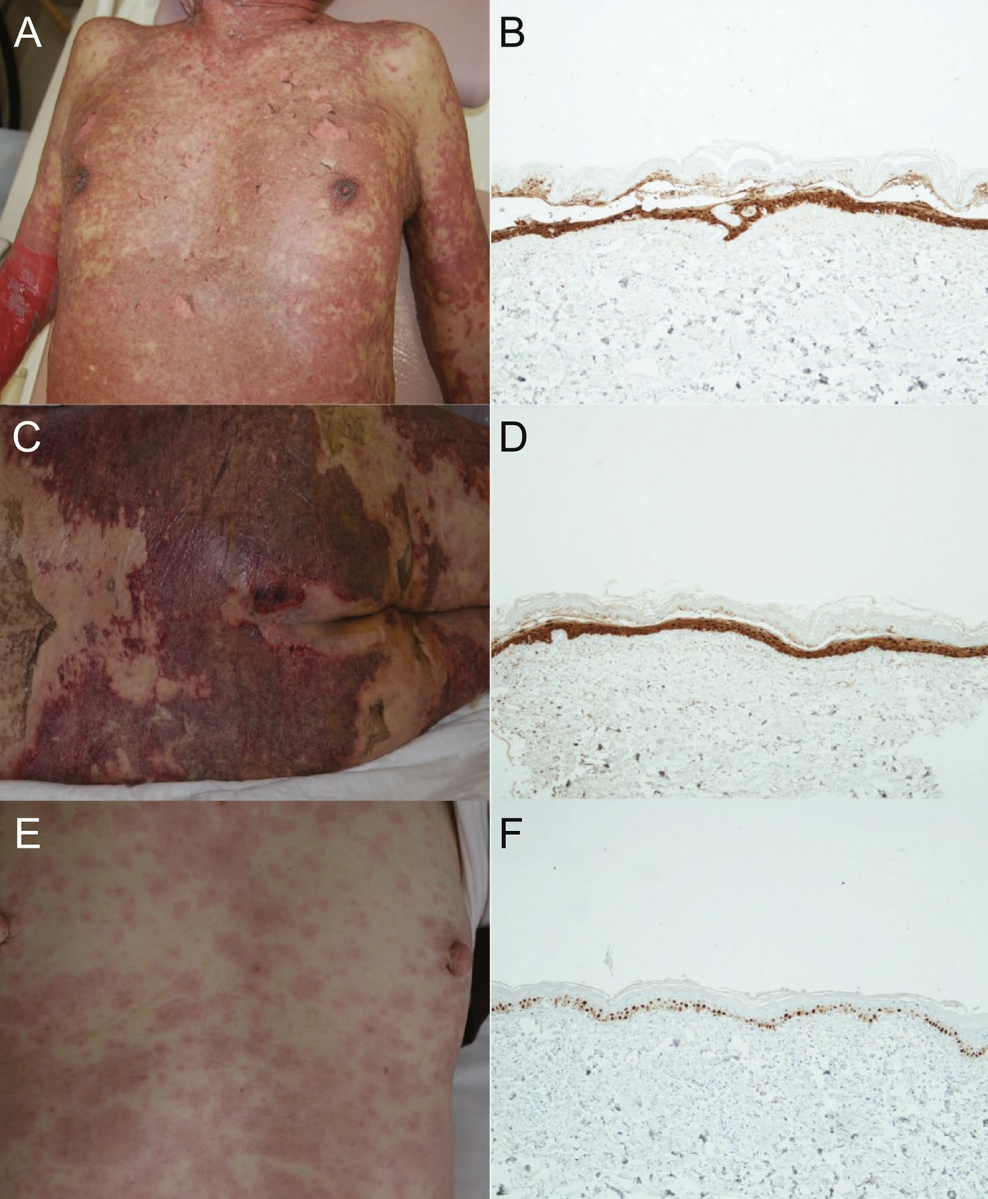
S100A2 expression in the epidermis of patients with drug eruption and clinical manifestations. (**A**) Clinical manifestation of a patient with toxic epidermal necrolysis after exposure to celecoxib and (**B**) the expression of S100A2 in the epidermis. (**C**) Clinical manifestation of a patient with toxic epidermal necrolysis in response to phenytoin and (**D**) the expression of S100A2 in the epidermis. (**E**) Clinical manifestation of a patient with macular-type drug eruption after celecoxib treatment and (**F**) the expression of S100A2 in the epidermis.

Epidermal S100A2 is involved in telaprevir-related drug eruption and TCE-stimulated keratinocytes. Analysis of a public microarray data set revealed that S100A2 expression was the maximum in the skin tissue (Supplementary Fig. S1). These findings prompted us to investigate the degree of involvement of S100A2 in drug eruption due to other medications. We performed immunostaining for S100A2 in the skin and evaluated the difference in its distribution in the epidermis between mild and severe forms of drug eruptions such as Stevens-Johnson syndrome and toxic epidermal necrolysis. Severe-type drug eruption in patients with toxic epidermal necrolysis due to phenytoin (Fig. 3A,B) and celecoxib (Fig. 3C,D) was related to high expression of S100A2 in the spinous layer of the epidermis. On the other hand, S100A2 expression decreased in the maculopapular type of drug eruption as compared with that in severe-type drug eruption and was limited to the basal layer of the epidermis (Fig. 3E,F).

To precisely evaluate the relationship between S100A2 expression and the severity of drug eruption, nine patients with severe-type drug eruption (Stevens–Johnson syndrome and toxic epidermal necrolysis) and 55 patients with mild-form drug eruption were enrolled in this study to clarify the degree of S100A2 expression in their skin (Table 2). The majority of the patients with mild-form drug eruption had limited expression of S100A2 within the basal layer of the epidermis, while 4 of 9 patients with severe-type drug eruption showed extended S100A2 expression into the spinous layer of the epidermis. These findings suggest that S100A2 expression may increase upon development of severe-type drug eruption.

**Table 2:** Comparison between maculopapular type and severe type drug eruption.

	Maculopapular type	Severe type	p-value
S100A2 expression			0.0364
Basal layer-dominant	44	4	
Basal layer and the spinous layer	11	5	

### S100A2 is involved in other inflammatory skin diseases

**Figure 4:**
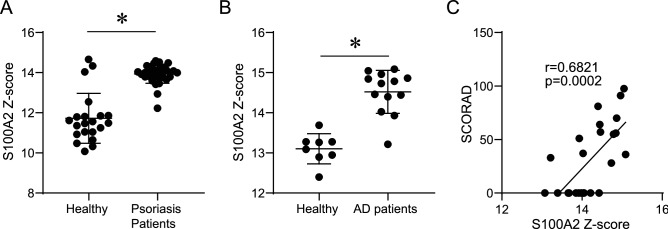
S100A2 expression in inflammatory skin diseases. S100A2 expression in the skin of patients with (**A**) psoriasis and (**B**) AD. Results are expressed as mean ± SD. (**C**) Correlation between SCORAD and the expression of S100A2. All p-values were obtained by the Student’s *t*-test; *p < 0.05.

Our results confirmed that telaprevir and TCE enhanced S100A2 expression in keratinocytes; whether S100A2 is a specific marker for drug-induced cutaneous inflammation is, however, unclear. We investigated S100A2 expression in cutaneous inflammatory diseases without telaprevir involvement. To ensure human relevance, we examined the mRNA expression of *S100A2* in other human skin diseases such as psoriasis and AD using public microarray data sets. Consistent with our study showing increased S100A2 production in the lesional skin of patients with severe drug eruption, the mRNA expression of *S100A2* was significantly higher in the lesional skin than in the healthy control skin (Fig. 4A,B). Consistently the SCORAD score, a scale of the severity of AD, was significantly and positively correlated with S100A2 expression (Fig. 4C), indicating that S100A2 is involved in representative inflammatory skin diseases. Thus, S100A2 is not a specific marker but a marker of keratinocyte damage in response to any inflammatory or toxic condition.

## Discussion

In this study, we first demonstrate the upregulated expression of S100A2 in keratinocytes of patients with drug eruption in response to telaprevir, which frequently causes severe drug eruptions. S100A2 expression was upregulated in patients with severe-type drug eruptions and other inflammatory skin diseases. These results suggest that S100A2 might not be a specific marker but serve as a plausible marker for keratinocyte-damaged skin inflammation.

Several studies have suggested the potential role of S100A2 as a biomarker. For instance, S100A2 expression loss serves as an independent prognostic marker in early-stage oral cancer patients at high risk of recurrence (14). S100A2 is also reported to be a potential marker of tumor progression or prognosis in pancreatic carcinogenesis and pancreatic cancer (15). It is imperative to discover useful biomarkers for severe-type drug eruptions, which are often life-threatening and unpredictable. Our study shows that severe-type drug eruption in response to telaprevir was associated with an increase in S100A2 expression in the epidermis. Furthermore, S100A2 overexpression in the epidermis was also observed in other representative inflammatory skin diseases and correlated with the severity of AD. Therefore, S100A2 may be a potential marker for keratinocyte damage owing to any inflammatory or toxic condition.

Drug-induced hypersensitivity nephritis is one of the biggest issues related to telaprevir treatment^29,30^. Among various organs, the kidney showed the second most abundant expression of S100A2 (Supplementary Fig. S1). LST is a useful diagnostic tool not only for drug eruptions but also for drug-induced nephropathy^31^; however, the results of LST were negative for all our patients. Thus, LST might not be useful for the diagnosis of telaprevir-induced hypersensitivity nephritis, which may involve S100A2. There might be a possible interaction between S100A2 and the immune system, which warrants further studies.

The actual impact of S100A2 on inflammatory skin diseases remains unclear; however, our public microarray data sets revealed that S100A2 is possibly involved in the pathogenesis of inflammatory skin diseases. We failed to elucidate the detailed effects of S100A2 on inflammatory cells in the skin. On the contrary, S100A2 might play a regulatory role in the inflammatory response by mediating a negative feedback effect on inflammatory skin diseases. Indeed, S100A2 regulates in vitro squamous cell carcinoma migration by exerting a favorable inhibitory effect on metastasis (10), indicative of its negative regulatory effects on the migration of some other cells. To clarify whether S100A2 has a positive impact on inflammatory skin diseases, keratinocyte-specific S100A2-deficient conditions should be examined under various inflammatory stimulation conditions.

In conclusion, S100A2 is highly expressed in the epidermis under inflammatory conditions and drug eruptions. Further investigation is necessary to clarify its role in cutaneous inflammatory skin diseases.

## Supplementary Information

Supplementary information.

## Data Availability

The microarray dataset is available at the National Center for Biotechnology Information’s Gene Expression Omnibus database (GEO accession: GSE154672 and GSE154713).
