# Supplementary material for: High S100A2 expression in keratinocytes in patients with drug eruption
Source: Sci Rep. 2021 Mar 9;11:5493. doi: 10.1038/s41598-021-85009-8 (PMC7943585; doi:10.1038/s41598-021-85009-8)
Supplement: Supplementary file 1 — Supplementary information. [file 41598_2021_85009_MOESM1_ESM.pdf]

High S100A2 expression in keratinocytes in patients with drug eruption

Manabu Yoshioka,[1] Yu Sawada,[1] Natsuko Saito-Sasaki,[1] Haruna Yoshioka,[1] Kayo Hama,[1] Daisuke Omoto,[1] Shun Ohmori,[1] Etsuko Okada,[1] Motonobu Nakamura[1]

1. Department of Dermatology, University of Occupational and Environmental Health

Corresponding author: Motonobu Nakamura, MD., PhD., and Yu Sawada, MD., PhD.
Department of Dermatology, University of Occupational and Environmental Health. 1-1 Iseigaoka, Yahatanishi-ku, Kitakyushu 807-8555, Japan
Tel: + 81 93 691 7445
Fax: + 81 93 691 0907
E-mail: motonaka@med.uoeh-u.ac.jp (MN) and long-ago@med.uoeh-u.ac.jp (YS)

Supplementary Figure 1

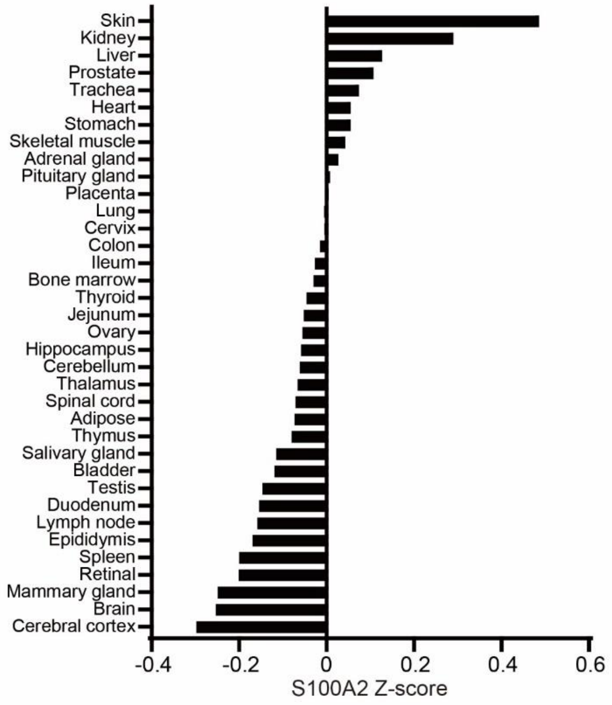

Supplementary Figure 1. S100A2 expression in various organs.

A public data set analysis shows the difference in S100A2 expression in various organs.
